# Pneumatocele With Perforation of the Residual Lung Immediately After Thoracoscopic Left Lower Lobectomy: A Case Report

**DOI:** 10.7759/cureus.81216

**Published:** 2025-03-26

**Authors:** Hiroyasu Matsuoka, Hirochika Matsubara, Mio Ota, Hiroyuki Nakajima

**Affiliations:** 1 Surgery, Kofu Municipal Hospital, Kofu, JPN; 2 Surgery, University of Yamanashi, Kofu, JPN

**Keywords:** lobectomy, lung cancer, perforation, pneumatocele, pneumothorax, pulmonary resection

## Abstract

Cases of pneumatocele formation following pulmonary resection are increasingly reported, yet its underlying pathogenesis and optimal treatment remain unclear. Here, we present a case of pneumatocele perforation occurring immediately after thoracoscopic left lower lobectomy. A 66-year-old man was found to have a suspicious nodule in the left lower lung zone during a routine chest X-ray as part of a medical checkup. CT suggested lung cancer in the left lower lobe, prompting referral to our hospital for further evaluation and treatment. Contrasted CT imaging revealed a ground-glass nodule with a maximum diameter of 4.6 cm, including a 4 cm solid component, located in segments S9/10 of the left lower lobe. No signs of emphysema were observed, and pulmonary function tests indicated normal respiratory capacity. PET-CT showed mild uptake (SUVmax 2.04) in the left lower lobe mass, with no evidence of distant metastasis. Additionally, contrast-enhanced brain MRI showed no abnormalities suggestive of metastasis. Bronchoscopy was performed, but transbronchial lung biopsy and brushing/irrigation cytology yielded no evidence of malignancy. Based on these findings, stage IB primary lung cancer was suspected, and a surgical biopsy followed by lobectomy was planned. The procedure was conducted using a four-port, completely thoracoscopic approach. Intraoperative needle biopsy confirmed adenocarcinoma, leading to left lower lobectomy and mediastinal lymph node dissection. Immediately after chest wound closure, a large volume of air leakage was observed, along with pneumatocele formation and perforation on the mediastinal side of the upper lobe. The pneumatocele was incised, its base cauterized, and covered with a TachoSil^®^ fibrin sealant patch (CSL Behring, King of Prussia, PA, USA). Postoperatively, a mild air leak persisted, requiring a single session of adhesive therapy. Lung fragility and increased negative intrathoracic pressure following resection are key risk factors for pneumatocele formation. While most cases can be managed conservatively, surgical intervention should be considered in symptomatic cases, particularly those presenting with pneumothorax or hemoptysis. If a pneumatocele develops intraoperatively, positive pressure ventilation and compression may cause further expansion. Therefore, we recommend prompt incision to prevent enlargement, followed by air leak closure through cauterization of the base and application of a sealing material.

## Introduction

Prolonged air leaks occur in 8-15% of patients following lobectomy [1], typically originating from the interlobar region or staple line, where suture closure or specialized coverage materials are used [2]. However, post-pulmonary resection pneumatocele formation leading to perforation and subsequent air leak is extremely rare, with only seven reported cases to date [3-9].

Currently, no consensus exists on the indications for surgical intervention or the optimal management of air leaks caused by pneumatoceles after pulmonary resection, making treatment particularly challenging. Here, we present a case of pneumatocele formation and perforation in the residual lung, detected immediately after lobectomy.

## Case presentation

**Figure 1:**
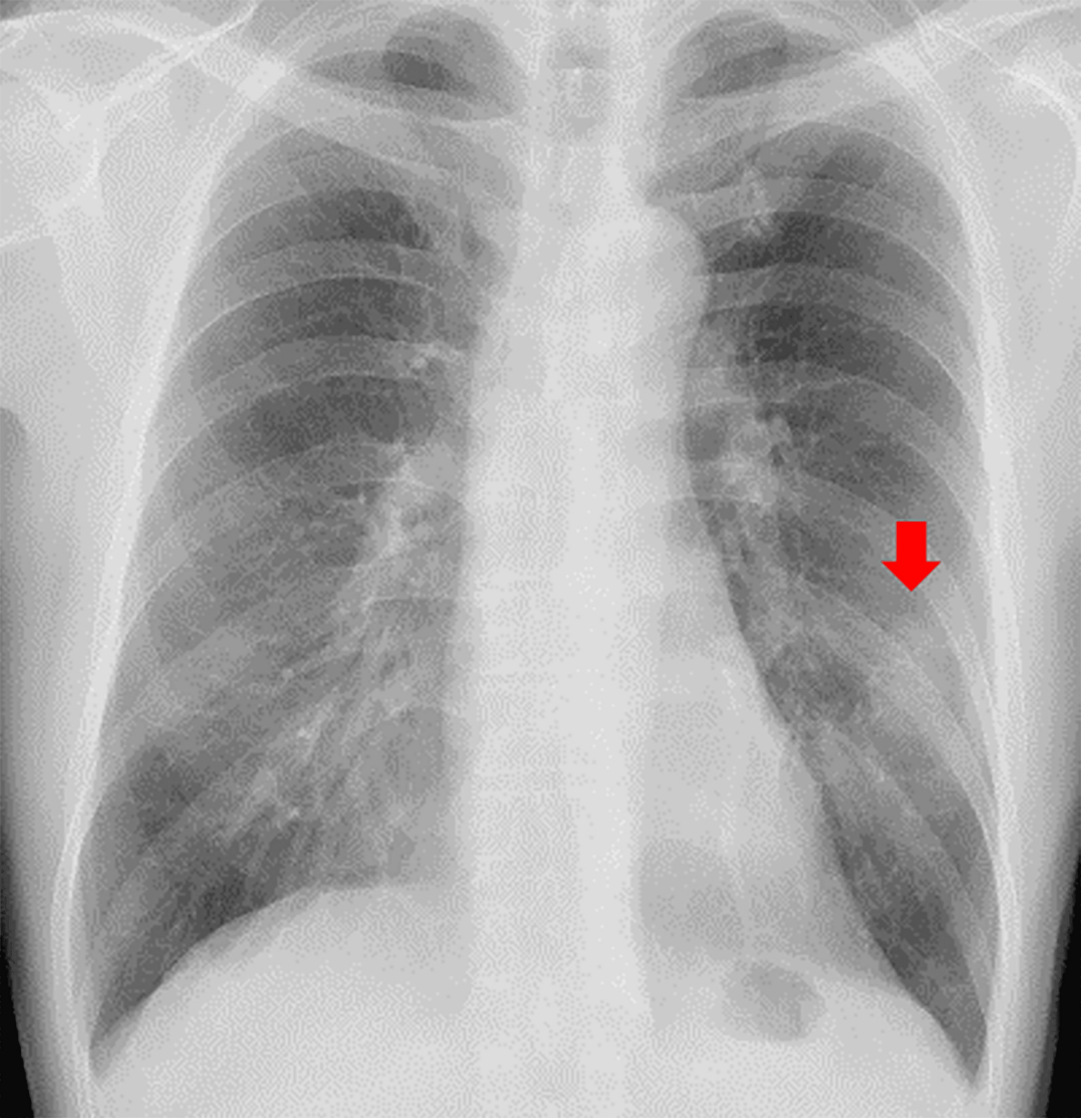
Chest X-ray findings The chest X-ray revealed a 3 cm mass in the left middle lung field.

A 66-year-old man underwent chest radiography during a routine medical checkup, which revealed an abnormality. The chest X-ray showed a 3 cm mass in the left middle lung field (Figure 1), leading to his referral to our hospital.

**Figure 2:**
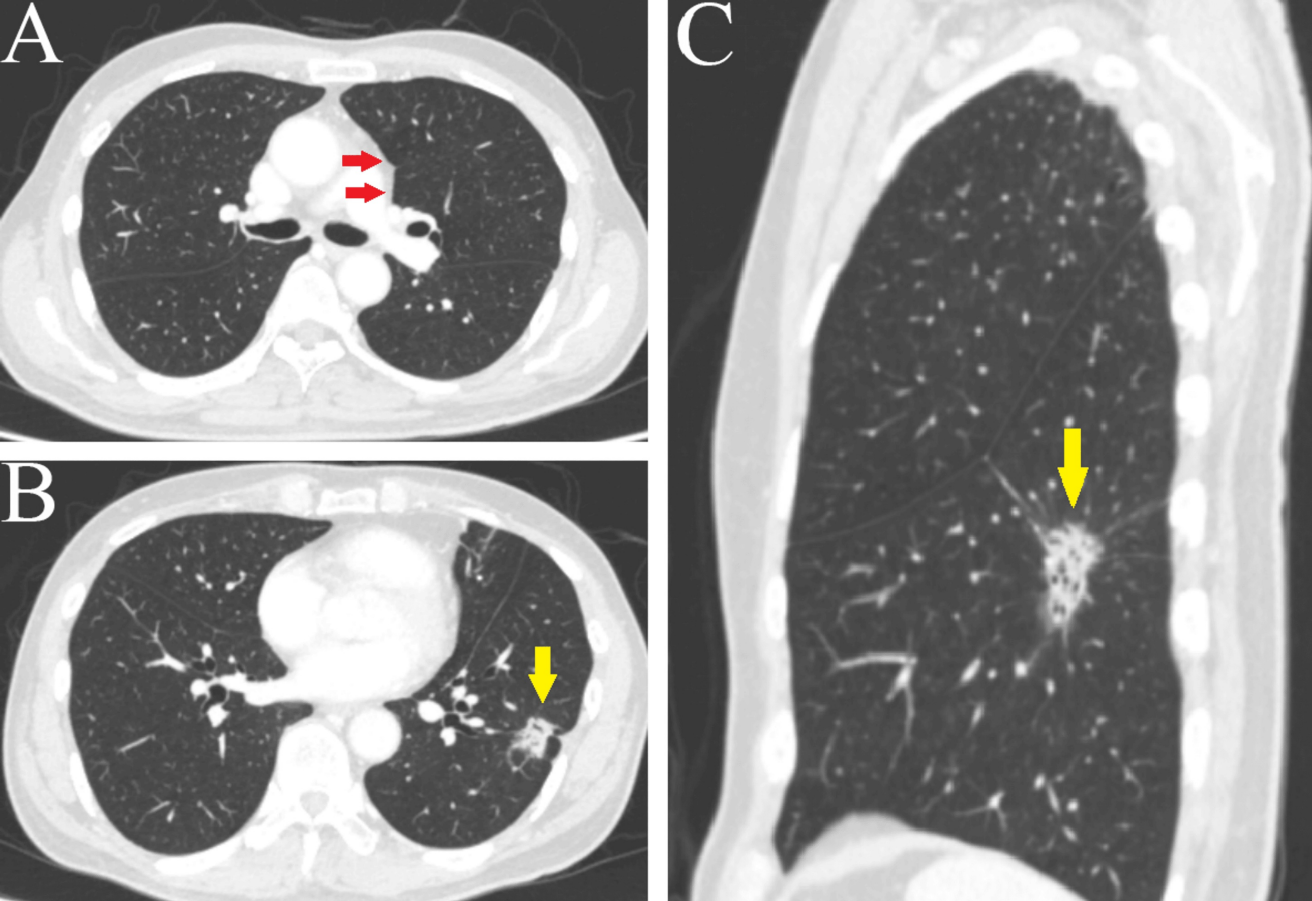
Contrast-enhanced CT findings (A) No emphysematous changes or inflammation were observed at the pneumatocele site (red arrow). (B) Horizontal view: a part-solid nodule measuring 1.6 × 1.4 cm, with a 1.5 cm solid component, was detected in S9/10 of the left lower lobe (yellow arrow). (C) Sagittal view: a part-solid nodule with a maximum diameter of 4.6 cm and a solid component measuring 4.1 cm was observed (yellow arrow).

The patient had a history of smoking 20 cigarettes per day until the age of 50 but had abstained for the past 16 years. His medical history included cerebral infarction, for which he had been taking 25 mg of clopidogrel before undergoing examinations and surgery. He also had hypertension, managed with 4 mg of candesartan. Blood tests revealed no significant abnormalities, and tumor markers were within normal ranges: carcinoembryonic antigen at 2.1 ng/mL, squamous cell carcinoma antigen at 1.5 ng/mL, and cytokeratin 19 fragment at 0.9 ng/mL. Contrast-enhanced CT imaging showed a ground-glass nodule with a maximum diameter of 4.6 cm and a solid component measuring 4 cm, located in S9/10 of the left lower lobe (Figure 2A, 2C). The imaging also confirmed the absence of emphysema (Figure 2B), and pulmonary function tests indicated normal respiratory capacity.

**Figure 3:**
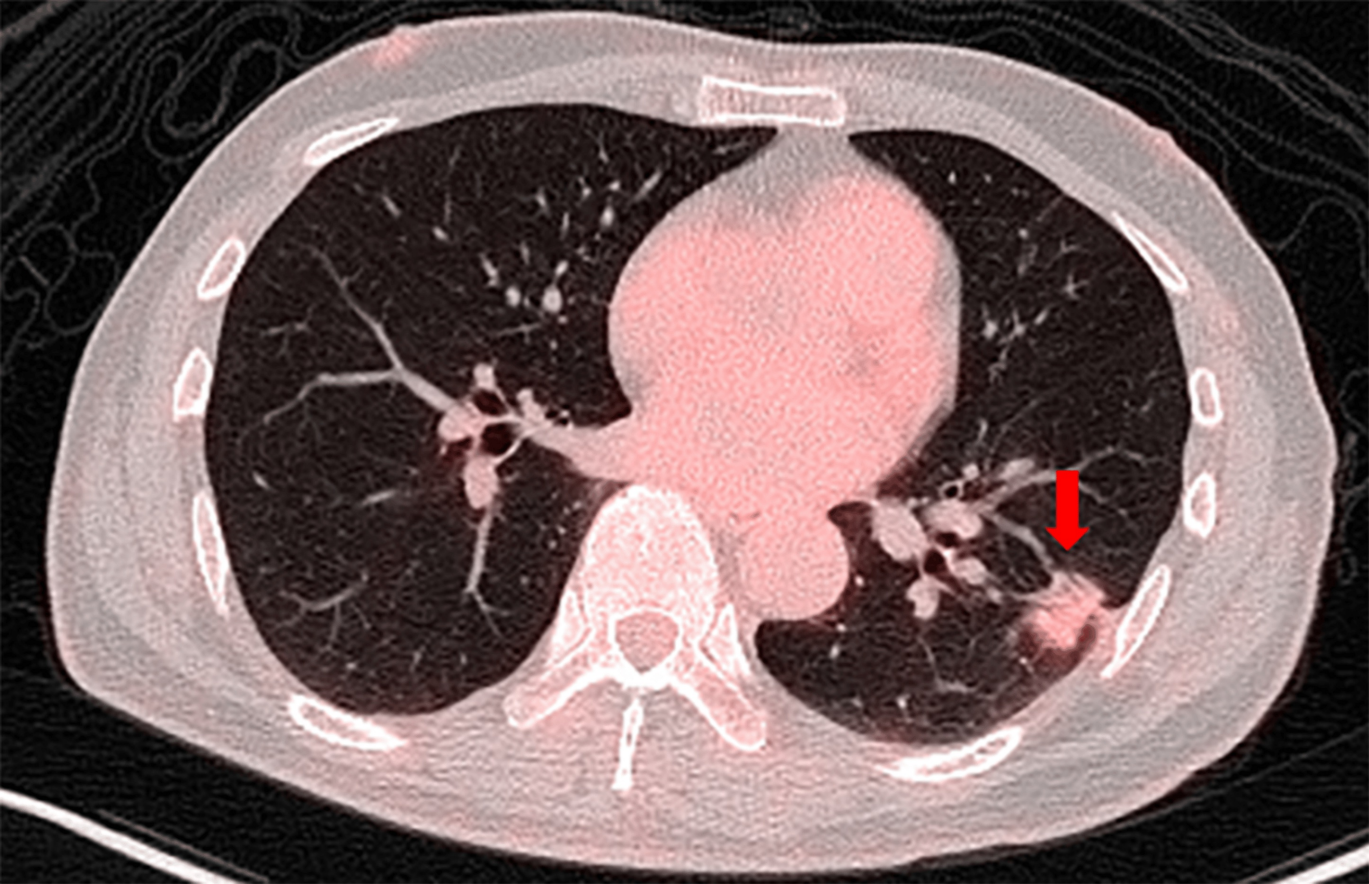
PET-CT findings Fluorodeoxyglucose accumulation was detected in the tumor in the left lower lobe, with an SUVmax of 2.04.

PET-CT revealed mild accumulation in the mass in the left lower lobe, with an SUVmax of 2.04. No evidence of accumulation suggestive of distant metastasis was observed (Figure 3).

Contrast-enhanced MRI of the head revealed no abnormalities suggestive of brain metastasis. Bronchoscopy was performed, but transbronchial lung biopsy and brushing/irrigation cytology results showed no evidence of cancer. Based on these findings, the patient was suspected to have stage IB primary lung cancer, and the plan was to perform a surgical biopsy followed by a lobectomy.

On the day of surgery, the patient’s temperature was 36.7°C, heart rate was 80 bpm, blood pressure was 140/80 mmHg, respiratory rate was 10 breaths per minute, and SpO₂ was 98%. Surgery was performed using a four-port thoracoscopic approach. Needle biopsy results confirmed adenocarcinoma. Due to the adhesion between the lingula and the mediastinum, adhesiolysis was performed. The pulmonary artery was exposed from the interlobar space, and the A6 and basal pulmonary arteries were divided using a stapler. The anterior part of the interlobar fissure was then divided with a stapler. The pulmonary ligament was divided to expose the inferior pulmonary vein, which was also divided using a stapler. The bronchus was taped and retracted anteriorly, followed by dissection of the #7 lymph nodes, which were then divided with a stapler.

**Figure 4:**
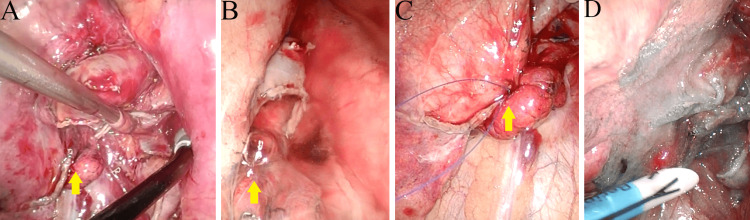
Intraoperative findings (A) Overall view after lobectomy, mediastinal nodal dissection, and identification of an air leak from the interlobe (arrow). (B) Closer view of the air leak from the interlobe (arrow). (C) Suture closure of the air leak (arrow). (D) Application of a polyglycolic acid sheet and fibrin glue reinforcement to seal the air leak.

The left lower lobectomy was performed according to the technique described by Bhende et al. [10]. After resection, a leak test was conducted, revealing an air leak at the anterior interlobar fissure (Figure 4A, 4B). The leak site was sutured, and a polyglycolic acid sheet with fibrin glue was applied for reinforcement (Figure 4C, 4D).

**Figure 5:**
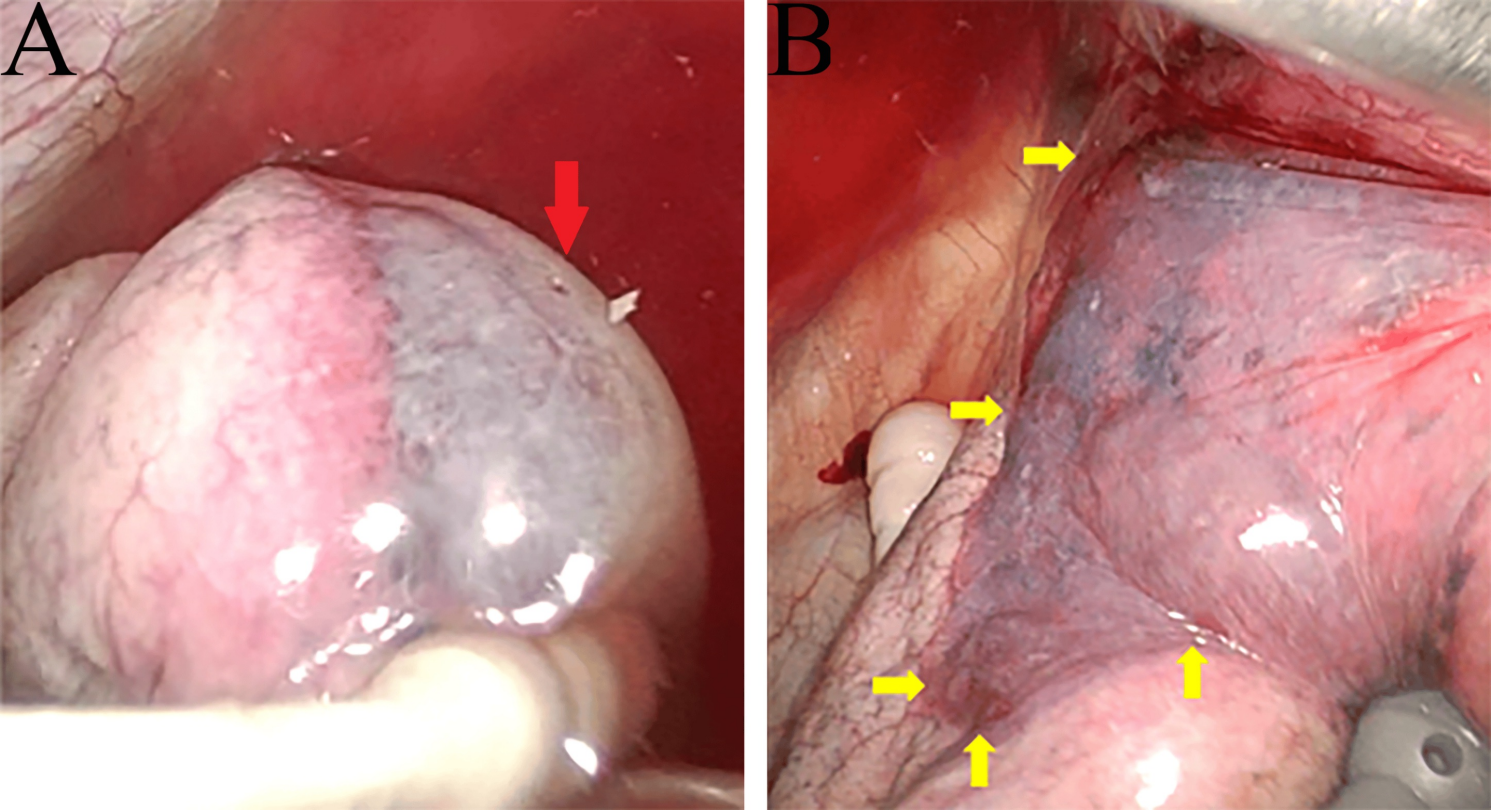
Intraoperative findings of the pneumatocele (A) Pneumatocele extending from the mediastinal to the lateral surface, exhibiting microperforation (red arrow). (B) Pneumatocele extending to the hilum, with margin expansion (yellow arrows).

After wound closure, continuous suction was initiated, revealing significant air leakage. No air leak was evident in the ventilator circuit before initiating continuous suction; however, once suction commenced, a distinct air leak became apparent. A port was placed for a leak test, which identified a pneumatocele on the mediastinal side of the upper lobe, accompanied by a perforation and associated air leak (Figure 5A). The pneumatocele extended near the superior pulmonary vein, and manipulation of the upper lobe led to further enlargement (Figure 5B).

**Figure 6:**
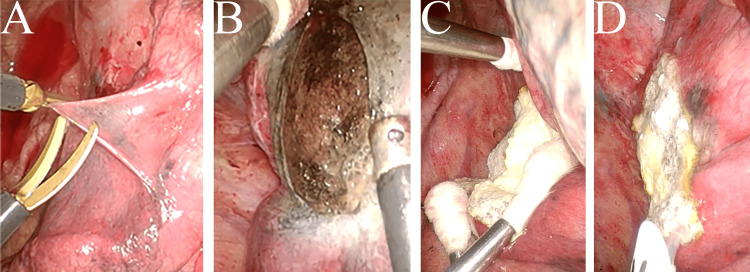
Surgical procedure for pneumatocele (A) Pleural incision at the pneumatocele site. (B) Cauterization of the pneumatocele base. (C) Application of a TachoSil^®^ fibrin sealant patch to the pneumatocele base. (D) Additional spraying of fibrin glue.

Given the risk of further expansion under positive pressure ventilation, the pleura was excised, and the lung parenchyma at the pneumatocele's base was cauterized using soft coagulation (Figure 6A, 6B). The affected lung parenchyma was then reinforced with a TachoSil^®^ (CSL Behring, King of Prussia, PA, USA) fibrin sealant patch and fibrin glue (Figure 6C). The surgery lasted two hours and 52 minutes, with an estimated blood loss too small to measure.

**Figure 7:**
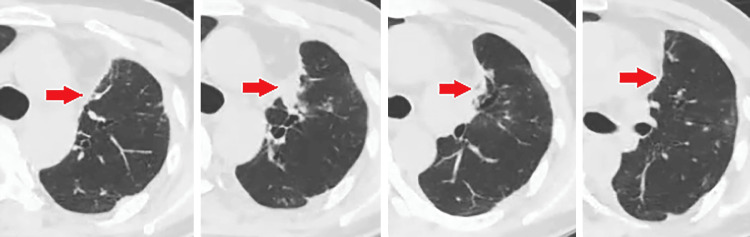
Postoperative contrast-enhanced CT findings Contrast-enhanced CT performed one year after lobectomy showed inflammatory changes at the pneumatocele site (arrow), but no pneumatocele was detected.

Postoperatively, a small residual air leak persisted, requiring adhesive therapy with a 50% dextrose solution. The drain was removed on the fifth postoperative day. A contrast-enhanced CT scan performed one year after surgery showed no evidence of cystic lesions (Figure 7). Since then, an additional two years and eight months have passed without any recurrence.

## Discussion

Pneumatoceles, also known as pulmonary pseudocysts, are thin-walled, air-filled cystic lesions that develop within the lung interstitium [11]. They are believed to form due to a bronchial one-way valve mechanism caused by trauma or inflammation or from the expansion of pseudocysts resulting from traumatic lung lacerations influenced by airway pressure or negative intrathoracic pressure. Additionally, factors such as inflammation-induced lung fragility, delayed healing due to corticosteroid use, persistent coughing, and positive pressure ventilation can contribute to pneumatocele expansion and perforation [11-13].

To date, only 12 cases of pneumatocele formation following lung resection have been reported, including this case. Among these, 10 cases presented with pneumothorax, while only two occurred intraoperatively (Table 1) [3-9].

**Table 1:** Reported cases of pneumatocele formation following pulmonary resection AE: acute exacerbation; COPD: chronic obstructive pulmonary disease; IP: interstitial pneumonia; LLL: left lower lobe; LU: left upper; LUL: left upper lobe; NA: not assessed; PGA: polyglycolic acid; RLL: right lower lobe; RUL: right upper lobe

Year	Author	Resected lung lobes	Risk factor	Time to discovery	Perforation	Surgical technique	
								2018	Honma et al. [3]	LU segment	COPD	Four days	+	Resection	
								2020	Fujibayashi et al. [4]	RUL	IP-AE, steroid intake	29 days	+	Intercostal muscle covering	
2020	Sugimura et al. [5]	LUL	NA	Intraoperative	+	PGA sheet + fibrin glue	
2021	Kondo et al. [6]	RUL	Smoking	One month	-	Soft coagulation + fibrin glue	
		LUL	None	Nine months	-	Soft coagulation + fibrin glue	
2022	Okita et al. [7]	RUL	Eosinophilic pneumonia, steroid intake	16 days	+	PGA sheet + suture of cyst wall	
2023	Yoo et al. [8]	LUL	NA	Nine days	+	Resection	
2024	Tanaka et al. [9]	RLL	IP	19 days	+	PGA sheet + fibrin glue + suture of cyst wall	
		RUL	COPD	17 days	+	PGA sheet + fibrin glue + suture of cyst wall	
		RLL	None	Two months	-	PGA sheet + fibrin glue	
		LLL	Clarinetist	Three months	+	PGA sheet + fibrin glue	
2025	Our case	LLL	None	Intraoperative	+	TachoSil + fibrin glue	

The formation of pneumatocele following lung resection is influenced by factors such as micro-lung injuries during surgery and increased intrathoracic negative pressure after resection, in addition to the previously mentioned risk factors [9]. In our case, although the patient had a history of smoking, there were no indications of parenchymal fragility, such as emphysema. However, during the initial adhesiolysis of the lingula, compression was applied to the area where the pneumatocele later formed, suggesting the possibility of micro-lung injury at that time. Additionally, a significant air leak was observed immediately after chest closure and the initiation of thoracic drainage, indicating that a combination of positive pressure from mechanical ventilation and negative intrathoracic pressure may have led to lung overinflation. These factors are believed to have contributed to pneumatocele formation.

No standardized treatment protocol exists for postoperative pneumatoceles. However, surgical intervention is recommended for traumatic pneumatoceles or those arising as complications of COVID-19 infection when the pneumatocele does not resolve, air leakage persists, or infection or hemorrhage occurs [12-14]. Additionally, a pneumatocele measuring 4-5 cm or larger poses a heightened risk of pneumothorax [13,14].

For pneumatocele formation after lung resection, surgical treatment is warranted when risk factors and symptoms are present. In cases such as this one, where the pneumatocele develops intraoperatively, positive pressure ventilation during mechanical ventilation poses a risk of rapid alveolar expansion, making immediate decompression essential to prevent further enlargement.

Surgical approaches depend on the size and location of the pneumatocele. When the base is small and distant from the hilum, partial resection is an option [3,8]. However, many pneumatoceles have broad bases, necessitating a surgical approach that preserves lung volume. In such cases, incising the cyst and suturing large air leaks at the base may be required [4]. For small air leaks, soft coagulation and the application of sealants and fibrin glue may be effective [5-7,9]. We used TachoSil^®^ as the covering material, although studies suggest no significant difference in air leak closure between TachoSil^®^ and polyglycolic acid sheets, indicating that either material may be equally effective [15]. In cases where the pneumatocele does not enlarge during surgery, suturing of the pneumatocele wall may also be effective [7,9].

Although this case involves an adult, there are reports of successful treatment of pneumatocele in pediatric patients using percutaneous drainage and fibrin glue injection via a catheter. This approach may be considered when surgical intervention is not feasible [16-18].

## Conclusions

We report a case of pneumatocele formation occurring immediately after lobectomy, which expanded due to positive pressure ventilation. Although post-pulmonary resection pneumatoceles are rare, they can develop as a result of micro-lung injury during surgery, in combination with positive-pressure ventilation and the negative pressure created by thoracic drainage, even in the absence of parenchymal fragility. Intraoperative pneumatoceles can rapidly enlarge under positive pressure ventilation, making prompt incision essential to prevent further expansion.
